# Higher Prevalence of Immunosuppression Among US Adults: Implications for Coronavirus Disease 2019 and Respiratory Pathogen Vaccinations

**DOI:** 10.1093/ofid/ofae415

**Published:** 2024-07-18

**Authors:** Yijia Li, Camille N Kotton

**Affiliations:** Department of Medicine, University of Pittsburgh, Pittsburgh, Pennsylvania, USA; Department of Medicine, Massachusetts General Hospital, Harvard Medical School, Boston, Massachusetts, USA

To the  Editor—Immunosuppression (IS) is a wide spectrum of conditions and affects the general health in certain groups of populations. With a steady increase in use of immunosuppressants, especially the biological agents, more people are at risk for severe infection, especially respiratory infections, that may lead to hospitalization and adverse outcomes [1]. A recent study based on the US Centers for Disease Control and Prevention's National Health Interview Survey (NHIS) data showed that 6.6% of US adults had self-reported IS [2], more than double the 2013 reported rate [3] and still higher than the reported 2012–2017 rate using a different database [1]. As infectious diseases physicians, we question: how would this information translate into clinical care?

The coronavirus disease 2019 (COVID-19) pandemic highlighted the vulnerability of individuals with IS. Our recent study highlighted that these individuals are disproportionately affected by COVID-19 [4]. It is paramount to proactively protect those with IS by providing appropriate immunizations per Advisory Committee on Immunization Practices (ACIP) recommendations. However, it remains unclear how we are doing in real life to protect them in terms of providing recommended vaccinations. To this end, we aimed to further evaluate the 2021 and 2022 NHIS surveys for the impact of self-reported IS on COVID-19–related metrics and vaccinations against other respiratory illnesses.

We adopted the same algorithm to categorize IS as described for previous NHIS reports [2, 3]. Briefly, anyone who answered “yes” to questions about “taking medications or treatments that would weaken the immune system” (variable name MEDRXTRT_A) or “medical conditions with weakened immune system” (variable name HLTHCOND_A) would be categorized in the IS group. In addition, those who answered “yes” to questions related to hematologic malignancy (variable names BLOODCAN_A, LYMPHCAN_A, and LEUKECAN_A) with dates of onset within 2 years of the survey [2, 3] were categorized in the IS group. Stata 17.0 and R (4.3.1) software were used for statistical analyses.

We found that the 2022 IS rate was further increased to 7.4% (weighted 95% confidence interval, 7.1%–7.8%; n = 27 651 surveyed) from 6.6% (6.3%–7.0%; n = 29 482 surveyed) in 2021 (all participants included). In 2021, 16.4% and 14.0% of participants in IS and non-IS groups, respectively, had COVID-19 (self-reported diagnosis and/or positive test results), while in 2022, 43.1% and 39.0% in IS and non-IS groups had COVID-19 (Figure 1 and Supplementary Table 1).

**Figure 1.**
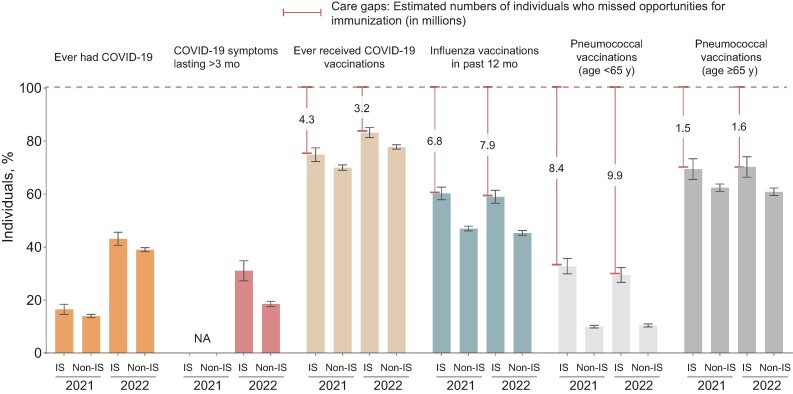
Coronavirus disease 2019 (COVID-19)–related metrics and respiratory pathogen vaccination rates. We also demonstrated care gaps in the population with immunosuppression (IS), denoted as the number of individuals with IS (in millions) who missed their opportunity to receive recommended vaccinations. Weighted prevalence and 95% confidence intervals are shown in the bar graph. Abbreviations: NA, not available.

To understand the potential care gaps in the IS group, we assessed COVID-19 vaccination rates, prolonged COVID-19 symptoms, and influenza/pneumococcal vaccination rates. In 2021, 74.9% and 70.0% of individuals in IS and non-IS groups, respectively, had received any COVID-19 vaccination; by 2022, these rates increased only incrementally, to 83.3% and 77.8%. Prolonged COVID-19 symptoms among individuals ever having symptomatic COVID-19 (available only in the 2022 survey) were disproportionately more common in the IS than in the non-IS group (weighted rates, 31.0% and 18.5%, respectively). For vaccinations against other respiratory illnesses, approximately 60% and 45%–47% of participants in IS and non-IS groups had received influenza vaccinations within 12 months at the time of the survey in both 2021 and 2022. Pneumococcal vaccination rates in IS groups were low, especially in individuals <65 years old, at about 30% in both years, despite ACIP recommendations [5] (Figure 1). These gaps correspond to missed opportunities for approximately 3–10 million individuals with IS for ACIP-recommended vaccinations (Figure 1).

Our analysis demonstrated the disproportionate impact of COVID-19 on individuals with IS, including higher rates of COVID-19 and long-lasting COVID-19 symptoms. We also highlight significant care gaps among individuals with IS, including a plateaued COVID-19 vaccination rate of approximately 80% and low influenza and pneumococcal vaccination rates.

Our study had several limitations. Analyses based on NHIS survey data are limited by the use of self-reported data and a lack of nuance as to the extent and heterogeneity of IS, which are variably associated with shedding of severe acute respiratory syndrome coronavirus 2 and COVID-19 outcomes [4]. Particularly, we could not obtain information on participants’ specific immunosuppressing conditions or the immunosuppressants they were taking. Moreover, the definition of IS used did not include other potentially immunocompromised individuals, including those with solid tumors receiving chemotherapy or immunotherapy. This survey also does not include enough black, indigenous, or other people of color compared with the composition of the general population and thus may underestimate gaps in care.

In summary, there continue to be significant healthcare gaps among individuals with IS. Future strategies to improve vaccination rate and engagement in care are urgently needed. An integrative approach, including community outreach, web-based vaccination decision tools for immunocompromised individuals, information distribution, and advocacy programs, may help vaccination uptake in this vulnerable population [6].

## Supplementary Material

ofae415_Supplementary_Data

## Supplementary Data

Supplementary materials are available at *Open Forum Infectious Diseases* online. Consisting of data provided by the authors to benefit the reader, the posted materials are not copyedited and are the sole responsibility of the authors, so questions or comments should be addressed to the corresponding author.

## Note

***Potential conflicts of interest*.** Y. L. is a topic editor for DynaMed, which is not related to the current work. C. N. K. is a member of the Advisory Committee on Immunization Practice at the Centers for Disease Control and Prevention and an author at UpToDate, both of which relate to vaccines. She is also a consultant for Takeda, Qiagen, Merck, Roche, Biotest, Evrys, and Exevir, none of which relate to the current work.
