# Supplementary material for: Higher Prevalence of Immunosuppression Among US Adults: Implications for Coronavirus Disease 2019 and Respiratory Pathogen Vaccinations
Source: Open Forum Infect Dis. 2024 Jul 18;11(8):ofae415. doi: 10.1093/ofid/ofae415 (PMC11295206; doi:10.1093/ofid/ofae415)
Supplement: ofae415_Supplementary_Data [file ofae415_supplementary_data.docx]

# **Methods**

Data were retrieved from National Health Interview Survey (NHIS) 2021 (<https://www.cdc.gov/nchs/nhis/2021nhis.htm>) and 2022 (<https://www.cdc.gov/nchs/nhis/2022nhis.htm>) as of February 19, 2024. We downloaded the publicly available ASCII data files from both years and pre-processed them using the recommended Stata input statements provided by NHIS. Variance estimation was performed using CDC’s recommended methodology using svy function (<https://ftp.cdc.gov/pub/Health_Statistics/NCHS/Dataset_Documentation/NHIS/2022/srvydesc-508.pdf> ) on Stata 17 SE platform (StataCorp) and svydesign function from the survey package on R (4.3.1). We used the default Taylor linearization for variance estimation and reported 95% confidence interval (CI). This analysis only involves a publicly available de-identified dataset and thus not considered human subjects research and does not require institutional review board review.

For COVID-19 vaccination, influenza vaccination and pneumococcal vaccinations, generally less than 5% of participants answered “Not Ascertained”, “Refused”, or “Don't Know”, and we impute those as not receiving the vaccinations. Care gap estimation was based on 2022 Census.gov data, available at <https://www.census.gov/quickfacts/fact/table/US>.

# **Data Sharing Statement**

All the data are de-identified and publicly available at the CDC websites <https://www.cdc.gov/nchs/nhis/2021nhis.htm> and <https://www.cdc.gov/nchs/nhis/2022nhis.htm>. Additional Stata do files to reproduce the results included in this analysis were uploaded at <https://github.com/yijiali89/NHISanalysis>.

**Supplementary Table**

| Supplementary Table 1. Demographics of survey respondents. | | | | |
| --- | --- | --- | --- | --- |
| Year | **2021** | | **2022** | |
|  | Immunocompetent  (N = 27,355) | Immunosuppression  (N = 2,127) | Immunocompetent  (N = 25,431) | Immunosuppression  (N = 2,220) |
| Age (years), n (%) |  |  |  |  |
| ≥18 to ≤64 | 19,129 (69.9) | 1,390 (65.4) | 17,422 (68.5) | 1,394 (62.8) |
| ≥65 to ≤84 | 7,176 (26.2) | 669 (31.5) | 7,025 (27.6) | 744 (33.5) |
| ≥85 | 968 (3.5) | 64 (3.0) | 921 (3.6) | 81 (3.6) |
| Refused to answer/unknown | 82 (0.3) | 4 (0.2) | 63 (0.2) | 1 (<0.1) |
| Sex assigned at birth, n (%) |  |  |  |  |
| Female | 14,723 (53.8) | 1,379 (64.8) | 13,663 (53.7) | 1,387 (62.5) |
| Male | 12,630 (46.2) | 748 (35.2) | 11,766 (46.3) | 832 (37.5) |
| Refused to answer/unknown | 2 (<0.1) | 0 (0.0) | 2 (<0.1) | 1 (<0.1) |
| Race, n (%) |  |  |  |  |
| AIAN and any other group | 238 (0.9) | 28 (1.3) | 194 (0.8) | 20 (0.9) |
| AIAN only | 214 (0.8) | 21 (1.0) | 251 (1.0) | 18 (0.8) |
| Asian only | 1,756 (6.4) | 75 (3.5) | 1,605 (6.3) | 83 (3.7) |
| Black/African American only | 3,048 (11.1) | 234 (11.0) | 3,019 (11.9) | 212 (9.5) |
| Other single and multiple races | 417 (1.5) | 18 (0.8) | 339 (1.3) | 19 (0.9) |
| Refused to answer/unknown | 1,392 (5.1) | 93 (4.4) | 1,323 (5.2) | 94 (4.2) |
| White only | 20,290 (74.2) | 1,658 (78.0) | 18,700 (73.5) | 1,774 (79.9) |
| Ethnicity, n (%) |  |  |  |  |
| Hispanic | 3,846 (14.1) | 235 (11.0) | 3,699 (14.5) | 244 (11.0) |
| Non-Hispanic | 23,509 (85.9) | 1,892 (89.0) | 21,732 (85.5) | 1,976 (89.0) |
| Insurance, n (%) |  |  |  |  |
| Covered | 25,024 (91.5) | 2,048 (96.3) | 23,344 (91.8) | 2,141 (96.4) |
| Not covered | 2,237 (8.2) | 76 (3.6) | 2,008 (7.9) | 75 (3.4) |
| Refused to answer/unknown | 94 (0.3) | 3 (0.1) | 79 (0.3) | 4 (0.2) |
| AIAN, American Indian or Alaskan Native. | | | | |
